# Why People Gamble: A Qualitative Study of Four New Zealand Ethnic Groups

**DOI:** 10.1007/s11469-012-9380-7

**Published:** 2012-04-14

**Authors:** Samson Tse, Lorna Dyall, Dave Clarke, Max Abbott, Sonia Townsend, Pefi Kingi

**Affiliations:** 1 School of Population Health, Faculty of Medical and Health Sciences, University of Auckland, Auckland, New Zealand; 2 School of Population Health, Faculty of Medical and Health Sciences, University of Auckland, Auckland, New Zealand; 3 School of Psychology, Massey University, Palmerston North, New Zealand; 4 Faculty of Health and Environmental Sciences, Auckland University of Technology, Auckland, New Zealand; 5 Faculty of Education, University of Auckland, Auckland, New Zealand; 6 NIU Development Incorporated, Auckland, New Zealand; 7 Department of Social Work and Social Administration, Faculty of Social Sciences, The University of Hong Kong, Pokfulam, Hong Kong

**Keywords:** Gaming, Ethnicity, Culture, Immigrants, Public health

## Abstract

In multicultural countries such as New Zealand, it is particularly important that gambling research take into account possible cultural differences. Many New Zealanders come from cultures that do not have a history of gambling, including the Mäori (New Zealand indigenous people), Pacific Islanders, and recent migrants. Little research has examined the reasons why people start and continue to gamble, especially among different ethnic groups. This research project thus aimed to develop a framework to explain how environmental, cultural, and social factors interact with personal attributes to determine gambling behaviors. In a qualitative study, 131 people broadly representative of Mäori, Pacific, Asian, and Päkehä/New Zealand European groups residing in New Zealand were interviewed individually or in focus groups. They included social and problem gamblers, families of problem gamblers, and professionals. Different personal, socioeconomic, environmental, and cultural factors were identified, summarized in a developmental framework, and compared to factors found for ethnic groups in other countries. Public health policy issues were raised, including greater control of gambling promotion.

## Background and Context

Historically, societies in many parts of the world did not gamble. Most parts of Australia and the South Pacific are among the regions where gambling was absent until the time of European colonization (Binde 2005). Since then, however, most societies have had some form of gambling. Moreover, during the past two decades many parts of the world have experienced unprecedented increases in gambling availability, participation, and expenditure. This growth has been particularly evident in countries such as New Zealand, Australia, and the Asia-Pacific region, where electronic gaming machines and large urban casinos have been widely introduced (Tse et al. 2010). These trends include a growing legitimacy and acceptance of gambling, the spread of gambling to previously non-gambling settings, the intersection of gambling and financial technologies, accelerated globalization, and the impacts of Internet gambling.

According to the latest census, the total population of New Zealand reached 4.4 million in 2011. A full 69 % of the population consists of persons of European descent, with the indigenous Mäori being the largest minority (14.6 %), followed by Asians (9.2 %) and Pacific peoples (6.9 %) (Statistics New Zealand 2011). The ethnicity of the population aged below 18 is more diverse (72 % European, 24 % Mäori, 12 % Pacific, and 10 % Asian) than those aged 65 years or older (91 % European, 5 % Mäori, 4 % Asian, and 2 % Pacific). The five largest cities are Auckland, Christchurch, Wellington, Hamilton, and Tauranga. Auckland has one third of the country’s population and is the most ethnically diverse city in New Zealand. Approval was given in 1990 for the establishment of casinos initially in Christchurch (1994) and in Auckland (1996), followed by four more in Queenstown, Dunedin, and Hamilton. The Gambling Act of 2003 provided an additional legislative framework to regulate and legalize gambling in New Zealand. Legalized gambling activities are believed to provide considerable funding support to an array of community, cultural, and sports groups. “For Maori, the indigenous population, it was proposed that these new casinos would provide employment and new opportunities for economic development” (Dyall et al. 2007, p. 85).

Abbott and Volberg (1999) have identified broad, interrelated contextual influences and trends that have shaped the evolution of commercial gambling internationally. Until the latter part of the 20th century, gambling was generally disapproved of, tightly regulated, and constrained in most Western societies. But a shift in attitudes toward gambling—particularly among the middle class—has helped to legitimize and legalize gambling in many parts of the world. Increased acceptance and availability have in turn led to gambling activities reaching further into societies and cultures, further advancing their acceptance and legitimacy. The spread of gambling to groups that previously had low levels of participation (women, for example) has been followed by increases in the prevalence of problem gambling in these groups. Thus, a feedback loop appears to be operating whereby public acceptance of gambling has contributed to further increases in gambling availability, which in turn has increased acceptance.

Historically, legal gambling was confined to a narrow range of settings. One of the most notable changes internationally has been the recent shift from gambling-specific venues to a wide variety of readily accessible social settings, such as family restaurants, entertainment centers, and holiday resorts, which previously were not associated with gambling (Petry 2006). This change is an aspect of gambling’s increasing integration with major social institutions, communities, and everyday life. These increases in the number, variety, and distribution of gambling venues—including the extension to previously non-gambling settings—have been referred to as “McGambling” (Goodman 1995) and “convenience gambling.” In addition to increasing physical accessibility, this extension reduces social and psychological barriers to access. Gambling becomes a backdrop in diverse environmental and social settings, reflecting and probably enhancing its widespread acceptance (or normalization).

Most people who gamble report that gambling is a satisfying and enjoyable activity, but some who gamble and their concerned significant others suffer from problem gambling (Mason and Arnold 2007). Many who gamble say they do so to win money, or else they think or dream about winning because it is fun and gives them pleasure, because it is a hobby or interest, because it is part of socializing with family and friends, and because it is exciting or relaxing (Abbott 2001). The generation of these positive mood states may well be a major reason many people continue to gamble even though they are aware they are likely to lose.

### Present Study

This research project explored why some people gamble by specifically examining the environmental, cultural, and social factors that interact with personal attributes to determine gambling behaviors. Moreover, in multicultural countries such as Aotearoa New Zealand, it is particularly important that gambling research takes into account possible ethnic differences.

## Method

This project was designed to take into account existing knowledge on gambling and the unique environmental, cultural, and social context of New Zealand in determining gambling behaviors. For such a method to be effective in evaluating these influences on gambling, we used a public health approach (for a general discussion, see Korn and Shaffer 1999; Volberg 1994). This approach views gambling as a product not only of biological and behavioral dimensions, but of broader population-level factors as well, including ethnicity, income, deprivation, employment, and poverty (Shaffer 2003). Another major methodological feature of this study was to collect data from New Zealand’s four main ethnic groups (Mäori, New Zealand European, Pacific peoples, and Chinese) and specific at-risk demographic groups, such as women and older people. Members of the project team were also academically and ethnically diverse, including investigators from three universities along with Mäori, Pacific Islanders, and Chinese researchers, so that we might implement this study in a culturally responsive manner.

### Participants and Recruitment

Ethics approval for the study was obtained from the University of Auckland Human Participants Ethics Committee (UAHPEC/346). An information sheet and consent form informed participants about the purposes of the survey, their rights as participants, the handling and anonymity of data, and the contact details of various ethnic problem gambling agencies in the event they felt distressed by the questionnaire. These forms clearly specified that all responses were to be anonymous and that no identifying details would be sought.

Four groups of people were recruited: people with gambling problems, people who gambled without problems, family members affected by problem gambling, and professionals working with gamblers. The individuals selected were broadly representative of the four main ethnic groups in New Zealand: Mäori, Pacific Islanders (Niue, Samoa, and Tonga), Chinese (migrated from China, Hong Kong, or Malaysia and residing in New Zealand less than 10 years), and Päkehä/New Zealand Europeans. “Päkehä” refers to people who are of European settler background.

Participants with problem gambling were recruited from specialized counseling services. People eligible to use free, government-funded services for problem-gambling treatment must meet the diagnostic criteria of problem/pathological gambling as measured by the South Oaks Gambling Screen (Lesieur and Blume 1987) or the Diagnostic and Statistical Manual-IV (American Psychiatric Association 1994). Those who self-identified as social or recreational gamblers in each ethnic group were recruited by the researcher in charge of that ethnic stream through his or her community network or cultural group. Family members affected by problem gambling were recruited through problem-gambling treatment services and general community networks. Professionals or counselors working in the gambling field were also recruited from problem-gambling treatment agencies. Altogether 131 people participated in the study, with each one taking part in either individual interviews or focus-group discussions. Table 1 shows the distribution of the participants in each category.

**Table 1:** Ethnic and other relevant backgrounds of research participants

	Mäori	Pacific Islanders	Chinese	Päkehä	Total
Problem gamblers	7 Ints *n* = 7	10 Ints *n* = 10	9 FGs, 7 Ints *n* = 16	2 FGs, 6 Ints *n* = 8	41
Family of problem gamblers	7 FGs, 1 Int *n* = 8	*n* = 0	4 FGs *n* = 4	*n* = 0	12
Recreational gamblers	10 FGs, 3 Ints *n* = 13	32 FGs, 5 Ints *n* = 37	3 Ints *n* = 3	4 Ints *n* = 4	57
Professionals	2 Int *n* = 2	11 FGs *n* = 11	2 FGs, 2 Ints *n* = 4	2 FGs, 2 Ints *n* = 4	21
Total	30	58^1^	27	16	*N* = 131

Figures denote the number of participants from each background

FG = focus group

Int = individual interview

*N* = total number of individuals interviewed in focus groups and in individual interviews

^1^The relatively large number of participants in the Pacific Islanders group reflects the different islands from which they originated

### Data Collection

Interviews and focus groups were conducted in either the participants’ preferred language or English, though notes were written in English only, and were audio-taped for future reference and cross-checking. The individual interview guideline consisted of three parts. In part one, the semi-structured interview began with questions about the person’s gambling behaviors (e.g., level of participation, severity of problems, and types of gambling) and what meaning the individual gave to gambling. Part two asked about how the individual started gambling. The third part contained questions about the individual’s gambling experiences.

This guideline was used to interview all four groups of participants, with slight modification to suit their particular background. For example, during interviews of practitioners, participating counselors were invited to provide their general observations instead of referring to individual clients. The focus-group discussion explored the link between sociocultural background and level of participation in gambling based on different structural factors (e.g., culture, gambling-related beliefs and assumptions, geographical location of residency, and local government policy on gambling) between ethnic groups (Korn and Shaffer 1999; Ministry of Health 2002; Schneiderman et al. 2001).

### Data Analysis

Data collection and analysis were concurrent and reflexive. Analysis began with the first interview or focus-group discussion. Each ethnic-specific researcher conducted the initial analyses and summaries of the information from the individual interviews and focus groups (except the practitioner focus group). Data were analyzed using a general inductive approach to identify key themes relevant to the research objectives (Thomas 2006). Concepts were reduced into themes and sub-themes and their linkages refined. Themes and sub-themes were developed by studying the written or transcribed data repeatedly. Special attention was given to possible meanings of each emerging theme or sub-theme, with new categories being created if existing themes did not encompass newly identified data from the interviews or focus groups. Research team meetings were held to monitor coding consistency and to ensure that the findings were supported by direct quotations from participant interviews.

Finally, we addressed the trustworthiness of the final analysis through triangulating data interpretation. The key findings were presented to members of the Mäori and Pacific National Reference Group and two problem-gambling counselors, who were asked to comment on their validity and the adequacy of analysis, and who confirmed that the findings reflected their experiences and perspectives well. In general, participants from different backgrounds—such as family members and people who gambled—had similar views on why people become gamblers, but the ethnicity-related differences were more complicated and will be elaborated in the Discussion section.

## Results and Analyses

On the whole, the interviews provided an opportunity to raise personal experiences, feelings, and thoughts about gambling experiences. In contrast, the focus groups provided in-depth material on wider environmental and sociocultural factors explaining why people gamble. Specifically, five major content themes were extracted from the participant discussions of the reasons why people gamble, as follows:

- Economic reasons, such as winning or close-to-winning experiences.
- Personal reasons, such as cognition, motivation for gambling, mental health, or mood.
- Recruitment (or retention) reasons, such as how gambling is normalized, encouraged, and promoted through advertising, consumerism, or government policy.
- Environmental reasons, such as the availability and accessibility of gambling activities, features of gaming machines, the gambling entertainment environment, and the Internet environment.
- Social reasons, such as the modeling of gambling behaviors and social participation with friends and family members who gamble.

### Economic Reasons

Most participants said they gambled because they expected to win money. Ongoing gambling was encouraged by positive memories, such as recollections of winning a prize, or by “close-to-winning” experiences. One participant said: *“I like to win. But it does not happen very often”* (Mäori, male, aged 30)*.* Financial reasons included the need for money to pay a debt or the potential of winning a big prize with a small amount of money; for example, the Lotto is a quick way to win money. One participant said that he gambled *“to get money mainly to pay my bills, mortgage, family needs”* (Mäori, male, aged 35). Problem-gambling counselors thought that Mäori people take up gambling mainly for socioeconomic reasons in that they are trying to “catch up” to the rest of society.

Niue social gamblers believed that people start gambling to win money for the family. They viewed it as an easy activity that could lead to financial gain or that served as a time-out from family. *“When my family has no money, sometimes I just try my luck”* (Pacific Islander, female, aged 26). For some Pacific Islanders, migration to New Zealand resulted in increased exposure to wealth and the placing of a monetary value on material things.

In contrast, according to Samoan focus-group participants, the main reason to begin gambling was poverty or low socioeconomic status. As one participant explained in detail:

**“** *We Tongan people as well as other PI [Pacific Islanders] came to New Zealand as a site or place of milk and honey. But now there is no milk and honey anymore. So we are looking for a new site or place within New Zealand for milk and honey and no wonder we have chosen gambling areas* **”** (Pacific Islander, female in focus group, aged 42).

Samoan interviewees felt that people start gambling to win money to help their family, pay bills, or give *fa’alavelave* (the traditional financial obligation to church or family), and so in general to ease financial problems. Likewise, according to the Tongan interviewees, people start to gamble because they need quick money for their bills, mortgage, or family. As one participant commented, it was *“to win some money, like I said earlier, to fulfill my dreams, like uplifting my family quality of life from poverty to riches”* (Pacific Islander, female, aged 54). Another Pacific focus-group member observed that people gamble because of a “lack of priority” when comparing themselves with other people’s material wealth, and gradually they develop “a sense of grandiosity” (as quoted by one practitioner) of wanting a big house, car, and other material possessions. Furthermore, among Pacific people money is always needed for church or family; for instance, *“for Samoans there are too many demands like fa’alavelave, church, work, or children”* (Pacific Islander, female, aged 52); also, there is *“pressure from the congregation to participate in fundraising—housie [bingo]; it starts out as fundraising and now becomes problematic”* (Pacific Islander, male in focus group, aged 52). In general, the group felt that the church’s acceptance of gambling behaviors was linked to the participation by individuals in gambling activities.

In comparison, European participants said they started gambling to win money or because they were in need of it. Sample comments included *“I wanted to win all the time”* (European, male, aged 28); *“I thought I could outsmart them”* (European, male, aged 60); *“Winning was exciting at first, later I was trying to recoup losses, so I bet more”* (European, female, aged 34).

The participants in the Chinese focus groups identified the chance to gain a *“big return by a small investment”* (Chinese, female in focus group, aged 40) as reasons why Chinese began to gamble. In the beginning, gambling is *“recognized as a kind of entertainment and most of the people can gain some winnings”* (Chinese, male, aged 24). Additionally, some Chinese people took up gambling because of low incomes compared with their earning capacity before migrating to New Zealand, seeing it as an opportunity to win money and regain status. Also, if unemployed, they had a lot of free time.

### Personal Reasons

A major personal reason to gamble was for excitement, relaxation, or “getting a rush.” According to participants, gambling was used as an escape mechanism from the depressing realities of their lives, stress resulting from moving to the city, and sometimes grief. One participant observed that *“their gambling behavior was a form of relief from depression”* (European, female, aged in 50s). Gambling was often perceived as an opportunity to improve quality of life, especially when boredom is constant.

Some Mäori participants said that gambling made it possible to be alone, and it could be a way to escape relationship problems. Samoan participants added that gambling was also a form of time-out, a release of stress from belonging to a large family. Similarly, Tongan participants said they also gambled to relieve boredom, as a break from housework, and as a form of socializing: *“To have a break from family issues . . . cooking, washing, etc., pokie machines take my mind away from family boredom issues”* (Pacific Islander, female, aged 55). One issue that appeared to be specific to Mäori people was the changing lifestyle of urban Mäori. Those who reside in urban areas, such as metropolitan Auckland, live fast-paced lives with long working hours. Also, more often than not, both parents in the family are working. Such a life is stressful, and managing stress is a priority. Many Mäori thus used gambling as a form of rest and relaxation.

For some Pacific Island peoples, a lot of stress came from being unemployed or having no money. In particular, *fa’alavelave* could become a financial burden, especially if someone was unemployed or *“not everyone [is] pulling their weight”* (Pacific Islander, female, aged 50). Another source of stress was boredom in the sense that some traditional activities were no longer available in New Zealand. The Pacific peoples’ focus group felt that people who were unemployed had more free time than those who worked, and so they would get bored and become tempted to gamble, especially on payday or benefit day.

Like the ethnic groups above, Asian participants saw gambling as a release from the stresses of work, allowing them to escape tough situations or relieve depressive feelings. As reasons to start gambling, participants cited immigration and post-immigration adjustment issues, such as communication problems, relationship problems, boredom, frustration, unemployment (or under-employment in some cases), and the absence of places to socialize and express themselves. As one participant vividly described: *“I gambled to find direction”* (Chinese, male, aged 43)*.* Some members said that New Zealand was boring and did not provide suitable entertainment for Asian people. Gambling was also a problem for international Chinese students studying in New Zealand who were without a proper role model. One group member elaborated: *“The parents of these children have always over-spoiled them or put too much pressure on these young generations who do not have good self-control”* (Chinese, female in focus group, aged 65).

### Recruitment Reasons

Between 50 % and 80 % of participants across the four ethnic groups could recall advertisings for Lotto, casino gambling, pokie (electronic gaming) machines, horse or dog racing, Keno, and Internet gambling. According to problem-gambling counselors from all four ethnic groups, advertising is a major influence on gambling, especially advertising for prizes and the high visibility of gambling opportunities in low socioeconomic areas. The practitioners in the group felt that the advertising was all positive in nature, for example, announcements of large jackpots or the opportunity to escape poverty. Advertisements portrayed city casinos as popular, safe, exciting, and glamorous places. One Mäori participant observed that in addition to advertising, alcohol played a role in the initiation of gambling. A Päkehä member in a focus group also pointed to alcohol as a trigger for gambling, giving the individual an *“excuse to go to the [gambling] venue”* (European, female, aged 34).

The Pacific Island practitioners additionally identified repetitive exposure to advertising as an important factor in initiating the gambling habit, such as sandwich boards located at congregation points. They viewed gambling venues, such as city casinos, as exciting and glamorous places to take family visiting from overseas. Another practitioner added that young people were often the targets of advertising, which was normalizing them to gambling at a very young age.

Asian participants also referred to new campaigns targeting specific ethnic groups, such as a city casino offering promotional deals to attract Asian people to gamble there. In a focus group, several participants also identified promotional materials used by casinos as an element that tempted self-barred patrons to continue going back.

### Environmental Reasons

In addition to advertising, gambling’s availability and prizes were given as other reasons to take up the habit. Gambling was a part of society; one participant observed that *“gambling supports society, while society supports gambling”* (Chinese, male, aged 33). Participants said that gambling activities were available in family restaurants that had side entrances so that young people could engage in them despite age restrictions. Pokies were particularly addictive, and even housie (bingo) led to problems. As one focus group member elaborated: *“Where money is a real issue for everyone, we’re being sold lotto [in shopping malls, supermarkets] and the dream of winning—it’s on TV all the time, network marketing”* (Mäori, female, aged 35). The variety of gambling facilities or the abundance of types of gambling was linked to the beginning of gambling, especially some activities that cannot be age-controlled, such as those involving toll-free numbers, text messaging, or Internet gambling. Other participants said that most gambling games were so easy to learn that they seemed to be tailored to different people’s skill levels and needs.

Other reasons included being brought up in an environment of gambling or being around people who gambled. Furthermore, in the past, *whänau* (extended family) gatherings (for example, birthdays and weddings) would often be held in the home of a *whänau* member or at the *marae* (Mäori meeting place). But currently such occasions were more likely to take place at venues for private functions, such as restaurants, pubs, or clubs. These new celebratory facilities usually had easy access to gambling.

For some immigrants, gambling was illegal in their home countries, and so its legality in New Zealand encouraged them to try it. Furthermore, casinos became popular destinations because people would see similar faces there, and so they felt safe and welcome. One participant said that the casino attracted a lot of Asian people because casino workers were well-trained and made patrons to feel welcome. Another participant elaborated: *“At a low tide in their lives, [casinos make them] feel like special, elegant people with their pleasant and polite greetings, VIP rooms, and consumer cards”* (Chinese, male in focus group, aged 60). An added reason for favoring casinos was that Asian people prefer table games to other forms of gambling.

### Social Reasons

The influence of the people around them—particularly friends and family—can also encourage gambling among individuals, such as *“hearing about other people often winning from pokies”* (Pacific Islander, male, aged 47). Family or friends can influence gambling behavior in two main ways: by initiating the gambling and by normalizing it. The participants in the Päkehä focus group felt that having a family background in gambling increased a person’s exposure to it and normalized such behaviors. Some Mäori focus group members outlined a “generational trend” of learned gambling behaviors and explained how they had been taught to use pokie machines by older members of their *whänau*: *“My dad and uncles played the horses; if I picked a winner I would get a lolly”; “It was normal for my whänau to bet on horses, housie, and cards”* (Mäori, male in focus group, aged 40). Among Asian people, the gambling behavior of family members and friends influenced participation: *“Ninety percent of my friends are gamblers”* (Chinese, male, aged 48). *“My friends convinced me to gamble. I had to show them I had money to save face”* (Chinese, male, aged 52). *“Those gambler friends called me to gamble; if I did not wish to go, they would say something that really harmed and challenged me, so that I had to bet for the sake of my face”* (Chinese, male, aged 41).

Gambling and the associated social environment could also be quite attractive for women. Gambling gave them something to do while also being a social occasion, a night out with “the girls” from the household. The Pacific/Samoan people enjoyed the social aspect of gambling: *“Just to relieve myself from boredom. To have a break from family issues like cooking, washing etc. [The] pokie machine takes my mind away from family, boredom”* (Pacific Islander, female, aged 45). In some cases Asian migrant women were employed in lower-skilled jobs and had not integrated into the larger society, and so they would look for others like themselves and find them at the casino. With regards to gambling among older Pacific peoples, participants emphasized that it was important to understand that grown-up children taking their parents out to dinner, to restaurants (where gambling machines are often present), or to the casino was *“a gift of love, a special treat”* (Pacific Islander, female in focus group, aged 60). For older Mäori, it was about recreation while meeting with other people. It was more of a hobby when there was nothing else to do and they were bored.

In some cases, gambling replaced people-to-people relationships. One Mäori participant described vividly how he liked engaging in an *“isolated relationship with the pokie machines”* (Mäori, male, aged 50) and did not like to be interrupted during this time.

## Discussion

Although different findings emerged from this study, the following discussion focuses on two specific areas. The first is the impact of the environment on gambling behaviors. Also, because a major aim of this project was to compare gambling experiences and patterns across four New Zealand ethnic groups, the second area of discussion covers culture and gambling.

### Environment and Gambling Behaviors

The last two decades in New Zealand have witnessed an increase in both the availability of gambling opportunities for individuals (such as the opening of casinos in Queenstown [two facilities], Dunedin, and Hamilton) and New Zealanders’ participation in gambling. As Abbott (2001) suggested: “Gaming machines play a particularly important role in the development of problem gambling, especially among women, and in diverse ‘mature’ gambling markets, they emerge as the dominant form in this regard” (p. 147). Pinge (2000) drew a very similar conclusion about the introduction of gaming machines in the Victorian city of Bendigo having significant negative economic and social impacts.

To date, most research explaining why people gamble and why some continue to do so at intense levels has focused on idiosyncratic, psychopathological motivations as well as the biological makeup of gamblers (e.g., Lee et al. 2007; Petry et al. 2003; Toneatto and Millar 2004). In contrast, one major finding from this study is the impact of environment on an individual’s gambling behavior.

In summation, this project outlines how the “environment” introduces people to gambling:

- Gambling activities promise attractive prizes;
- Gambling advertising targets specific ethnic or community groups (Dyall et al. 2007);
- An abundance of variety in gambling activities or machines caters to different skill levels;
- Accessibility to gambling venues is non-stop (24 h, 7 days a week);
- Families initiate and normalize gambling;
- Gambling is introduced by workmates and friends.

This understanding of gambling and its relationship to the environment resonates with the public-health approach to gambling. This approach views gambling in the environment itself, taking into account the level of gambling activities available in the area and the associated advertising (Dyall et al. 2007). Contemporary public health perspectives are not limited to the biological or behavioral dimensions, but instead also address socioeconomic determinants such as income, employment, poverty, and access to social and healthcare services related to gambling and health (Shaffer and Korn 2002).

### Ethno-cultural Perspectives on Gambling Behaviors

This is the first qualitative gambling study in New Zealand to examine gambling behaviors across four ethnic groups in a single study (see Cultural Partners Australia Consortium 2000, for similar work conducted in Australia). Data obtained from the four ethnic groups presented a snapshot of the responses from participants in each group. For example, some Mäori participants gambled for socioeconomic reasons, such as for money to meet their everyday needs. According to some participants, stressful city living encouraged them to gamble or gamble intensely. Another theme was the presence of gambling activities in celebratory venues. Pubs and clubs where people go to drink also have gambling facilities. Individuals in low socioeconomic areas are targeted by a high concentration of electronic gaming machines. Furthermore, a “generational trend” exists, whereby gambling is passed down to the younger generation—for example, young children are taught to gamble by their *whänau* (extended family). On the whole, gambling is becoming a part of Mäori community and social activities, such as the gambling activities located in *marae* (Mäori meeting places).

We acknowledge that these snapshots merely suggest the range of variation and do not fully address the interrelationships between gambling and culture. Most importantly, the results should be read with caution with respect to the enormous variations *among and within* these four ethnic groups. Another qualitative study (Tepperman et al. 2003) conducted in Canada investigated gambling behaviors across six ethnic groups: Aboriginal, British Isles, Caribbean, Chinese, Latin American, and Russian. Comparing the results of the Canadian study with the findings of this project shows that Aboriginal peoples in Canada and New Zealand indigenous people both saw gambling as a possible way out of poverty, with Aboriginals citing “the centrality of poverty and the belief that gambling is the only possible way to get out of it and escape from boredom and other addictive behaviors” (Tepperman et al., p. 81). When people from the British Isles—equivalent to Päkehä in this study—were asked why they gambled, their answers suggested a variety of reasons similar to the Päkehä responses, including the need for excitement, relief from stress and poverty, and the fact that gambling was an addictive behavior.

One noticeable difference between the Asian participants in this study and the Asian respondents in the Canadian study is that the latter commented on how gambling at home (e.g., playing cards or mahjong) differed from that in a public facility like a casino. In this study, the Asian or Chinese participants did not mention private gambling at all. But Asians in Canada and New Zealand face similar post-migration adjustment difficulties such as language barriers or a boring life in their new host country, and so “gambling in the casino, therefore, seems a possible way for the gamblers to put aside the problems facing them” (Tepperman et al*.* 2003, p. 95).

Raylu and Oei (2004; see Loo et al. 2008, 2011, for a comprehensive review of Chinese and gambling) have offered several reasons to explain the elevated rates of problem gambling among indigenous people and immigrants. First, gambling may be more available than in their country of origin, or they may be targeted by gambling promotional activities. Second, gambling changes its meaning when people move to another country; for example, it may be seen as a legitimate way to get “quick, easy money.” Third, gambling is used as a coping mechanism to deal with difficulties while trying to adapt to the mainstream culture. Fourth, and most ironically, the increased level of problem gambling among indigenous people and Asian immigrants might be related to a successful acculturation process. In other words, those newcomers who try to integrate with mainstream culture take up gambling because it is common, accepted, accessible, and liberalized in the host country, such as New Zealand or Australia.

Despite their being more likely to have gambling problems (Abbott and Volberg 1996, 2000; Abbott et al. 2004), research has indicated that Mäori, Pacific peoples, and Asian people may be less likely to seek help for such problems (Ministry of Health 2008; Wong and Tse 2003). One explanation for this is that “like Mäori, Pacific-oriented support services have been poorly resourced or completely un-resourced until very recently, although Pacific demographics in New Zealand have always suggested a high susceptibility to problem gambling” (GamblingWatch 2003, p. 34). Another possible explanation is the shame associated with problem gambling (Raylu and Oei 2004). “Losing more money than what one can afford and thereby jeopardizing the future prospects of one’s family in a new country leads a person to experience intense shame, devastating remorse, and the feeling of being a total failure” (Tse et al. 2004, p. 8). Perceptions and beliefs about problem gambling intervention programs (which consist primarily of counseling and psychotherapy) may also influence the level of service utilization: “It is also possible that gambling treatments, which are based on Western models, are not sensitive enough to address the needs of ethnic minorities and indigenous communities” (Raylu and Oei 2004, p. 1098).

## Conclusions

This study was an ethnic-based, cross-sectional examination involving members of the Mäori, Pacific, Päkehä, and Asian communities in New Zealand. It has provided a wealth of information about how study participants started to gamble, their gambling experiences, and the specific context of individual experiences. The findings highlight the different personal, socioeconomic, and cultural factors identified in each study group in relation to their gambling behaviors.

Like most research studies, this project is subject to several limitations and qualifications. In particular, the data the participants provided were retrospective and probably subject to the problems of response bias and faulty recall. Some participants might have been unaware of the influence of their own thinking (e.g., “inflated chance of winning,” illusion of control) on their reasons for taking up gambling.

Two recommendations emerge from this study. First, because a large number of participants commented that they were drawn (some said they were targeted) by advertising or promotional materials to gamble and subsequently developed problem gambling, it must be considered whether certain at-risk groups (including young people, Päkehä, Mäori, and Chinese students) are being targeted at a disproportionate level. Another important consideration is whether the product is presented fairly and accurately, given the addictive elements and potential harm caused by gambling. And second, some participants in this study said they started gambling because gambling venues were easily accessible and plenty of gambling options were available to cater to different interests and abilities. This particular finding raises the issue of the need to regulate the distribution of gambling activities (e.g., location and type of gambling outlets).
